# Supplementary material for: A high-resolution mRNA expression time course of embryonic development in zebrafish
Source: eLife. 2017 Nov 16;6:e30860. doi: 10.7554/eLife.30860 (PMC5690287; doi:10.7554/eLife.30860)
Supplement: Supplementary file 6. [file elife-30860-supp6.zip › biolayout-clusters-files/Cluster117-genes.html]

Cluster117


# Cluster117: Genes

| Ensembl ID | Gene Name | Chr | Start | End | Biotype |
| --- | --- | --- | --- | --- | --- |
| ENSDARG00000013711 | PLIN3 | 8 | 19240444 | 19250439 | protein\_coding |
| ENSDARG00000101190 | cyp2k8 | 3 | 12633011 | 12650931 | protein\_coding |
| ENSDARG00000039677 | dsc2l | 20 | 7272555 | 7303958 | protein\_coding |
| ENSDARG00000022309 | dspa | 2 | 1767465 | 1819985 | protein\_coding |
| ENSDARG00000000212 | krt97 | 22 | 11726358 | 11729424 | protein\_coding |
| ENSDARG00000036820 | mgll | 8 | 26273421 | 26307362 | protein\_coding |
| ENSDARG00000040309 | pxdc1b | 24 | 11310897 | 11327101 | protein\_coding |
| ENSDARG00000044251 | rasgef1bb | 10 | 9325720 | 9351108 | protein\_coding |
| ENSDARG00000079372 | si:ch211-264f5.6 | 19 | 5813597 | 5851476 | protein\_coding |
| ENSDARG00000030775 | sybl1 | 14 | 8727301 | 8750053 | protein\_coding |
| ENSDARG00000056618 | xk | 11 | 29571215 | 29586524 | protein\_coding |
